# History of Cyclodextrin Nanosponges

**DOI:** 10.3390/polym12051122

**Published:** 2020-05-14

**Authors:** Ilona Krabicová, Silvia Lucia Appleton, Maria Tannous, Gjylije Hoti, Fabrizio Caldera, Alberto Rubin Pedrazzo, Claudio Cecone, Roberta Cavalli, Francesco Trotta

**Affiliations:** 1 Department of Nanochemistry, Advanced Technologies and Innovation, Technical Institute for Nanomaterials, University of Liberec, Studentská 1402/2, 461 17 Liberec, Czech Republic; ilona.krabicova@tul.cz; 2 Department of Chemistry, University of Torino, via P. Giuria 7, 10125 Torino, Italy; silvialucia.appleton@unito.it (S.L.A.); maria_tannous@hotmail.com (M.T.); gjylije.hoti@unito.it (G.H.); fabrizio.caldera@unito.it (F.C.); alberto.rubinpedrazzo@unito.it (A.R.P.); claudio.cecone@unito.it (C.C.); 3 Department of Drug Science and Technology, University of Torino, via P. Giuria 9, 10125 Torino, Italy; roberta.cavalli@unito.it

**Keywords:** history, cyclodextrin nanosponge, crosslinked polymer

## Abstract

Nowadays, research in the field of nanotechnology and nanomedicine has become increasingly predominant, focusing on the manipulation and development of materials on a nanometer scale. Polysaccharides have often been used as they are safe, non-toxic, hydrophilic, biodegradable and are low cost. Among them, starch derivatives and, in particular, cyclodextrin-based nanosponges (CD NSs) have recently emerged due to the outstanding properties attributable to their peculiar structure. In fact, alongside the common polysaccharide features, such as the presence of tunable functional groups and their ability to interact with biological tissues, thus giving rise to bioadhesion, which is particularly useful in drug delivery, what makes CD NSs unique is their three-dimensional network made up of crosslinked cyclodextrin units. The name “nanosponge” appeared for the first time in the 1990s due to their nanoporous, sponge-like structure and responded to the need to overcome the limitations of native cyclodextrins (CDs), particularly their water solubility and inability to encapsulate charged and large molecules efficiently. Since CD NSs were introduced, efforts have been made over the years to understand their mechanism of action and their capability to host molecules with low or high molecular weight, charged, hydrophobic or hydrophilic by changing the type of cyclodextrin, crosslinker and degree of crosslinking used. They enabled great advances to be made in various fields such as agroscience, pharmaceutical, biomedical and biotechnological sectors, and NS research is far from reaching its conclusion. This review gives an overview of CD NS research, focusing on the origin and key points of the historical development in the last 50 years, progressing from relatively simple crosslinked networks in the 1960s to today’s multifunctional polymers. The approach adopted in writing the present study consisted in exploring the historical evolution of NSs in order to understand their role today, and imagine their future.

## 1. Introduction

Cyclodextrins (CDs) are natural oligosaccharides widely used in numerous fields, including biomedicine, cosmetics, food industry, wastewater remediation and catalysis. The popularity of CDs is mainly attributable to their inclusion capacity and ability to improve desired physico-chemical properties of guest molecules, such as apparent solubility and stability.

However, native CDs have some limitations, among all solubility. Time-consuming and expensive separation techniques would be necessary to recover the CDs from an aqueous environment. When CD polymers came on the scene, this limit was overcome and their solubility could be tuned by changing the degree of crosslinking. Nowadays, CD insoluble polymers are usually called “nanosponges” (NS), referring to their sponge-like structure, which has high porosity and capacity of entrapping various kinds of molecules into the matrix [1].

Being insoluble is not the only advantage of CD NSs. In fact, while internal cavities of CDs can host hydrophobic molecules, the interstitial pores present between crosslinker units and the external walls of CDs make NSs capable of also entrapping hydrophilic molecules [2,3]. This ability has triggered extensive research, and CD NSs have emerged as a promising material in various fields such as environmental, enzymological, agricultural, biomedical, catalytical and pharmaceutical applications, as well as in gas storage, flame retardants, etc. [4,5,6,7,8,9], which will be discussed in depth in this review.

Today’s main field of investigation is nanomedicine due to the target reached by nanoparticles as drug delivery systems [10]. Nanoparticles can be prepared using polymers, which must at least be biocompatible and are better if biodegradable. Use has been made of many different polymeric materials, such as polylactic and polyglycolic acids, polycaprolactone, polyacrylic acids, proteins, polypeptides (gelatin) and polysaccharides, which include CD NSs [10].

The advantages of using polysaccharides are that they can be easily modified chemically due to the presence of derivable groups on the molecular chains, and they are safe, non-toxic, hydrophilic, biodegradable and are low cost as they are readily available in nature. In addition, the hydrophilic groups contained in most natural polysaccharides, such as hydroxyl, carboxyl and amino groups, which may form non-covalent bonds with biological tissues (mainly epithelia and mucous membranes), thus giving rise to bioadhesion, prolong the residence time of the encapsulated drug and solve the bioavailability problems. These properties combined with the capability of NSs to carry a wide variety of drugs make NSs the therapeutic nanocarriers of choice.

A comprehensive overview of the current state of these NS drug delivery systems is provided in the review conducted by Caldera et al. [11], in which they were classified into four generations.

The 1st generation comprises urethane, carbonate, ether and ester NSs synthesized by reacting CDs with a crosslinking agent. In the 2nd generation, there are polymers with specific properties, e.g., fluorescence or charged side chains. The 3rd generation contains stimuli-responsive NSs modifying their behavior according to changes in the environment, such as pH, temperature gradients or oxidative/reducing conditions. The 4th generation includes molecularly imprinted NSs with high selectivity towards specific guest molecules.

NSs have proven capable of keeping up with the advances in nanomedicine, responding positively to the need for targeted treatments, aimed at improving the efficacy and reducing the adverse effects of the drugs. This approach is called “targeted drug delivery” and consists of assisting the drug molecule to preferably reach the desired site (cell/tissue/organ) [12]. Much consideration has, therefore, been given in pharmaceutical research to nanoparticles, including NSs, able to perform such delivery. The potential of nanosponges, in particular, is attributable to the presence of functional groups on their surface, which has led to their being recently used for grafting biological ligands capable of binding to specific receptors on the surface of the target cells, thus increasing cellular uptake of the drug encapsulated inside the NSs [12] and, consequently, the possibility of improving therapeutic efficacy.

These NSs functionalized with biological ligands may be considered as belonging to a new, fifth generation.

Today’s NSs are the result of intense research conducted over the years. They have progressed from the relatively simple crosslinked networks of the 1960s to today’s multifunctional polymers.

The synthesis of NSs over the years has evolved towards greener processes, starting from the use of organic solvents, which made the washing step of NSs necessary because of the toxicity, passing through water to the most recent solvent-free synthesis [13]. NS formulations have also been optimized by means of mathematical tools to ensure the best solution for the delivery of drugs [14,15,16,17]. The number of analytical tools has increased in order to improve the characterization of NSs and better understand their properties and behavior.

The purpose of this review is to provide an overview of research on CD NSs, by exploring their historical development to make the reader aware of how they were developed and used to meet the changing needs of the period, and highlighting their properties and enormous potential for the future.

We believe that studying the history of nanosponges is important because it allows us to understand the past, which, in turn, enables us to understand the present and possibly have a glimpse of the future opportunities of such innovative and promising polymers. As Winston Churchill said, “The farther backward you can look, the farther forward you are likely to see”.

## 2. Origin and Historical Development of Nanosponges Over the Last 50 Years

This review describes in detail the research related to crosslinked insoluble CD polymers from their origin to today’s multifunctional polymers.

### 2.1. From the 1960s to the 1980s: Origin of Insoluble Crosslinked Cyclodextrin Polymers

The history of crosslinked insoluble CD polymers dates back to 1965 when Solms and Egli published a study on the preparation and inclusion properties of novel network polymers made up of CDs crosslinked with epichlorohydrin (EPI). Firstly, CDs dissolved in water were activated with sodium borohydride and hot solution of sodium hydroxide, and then EPI was added as a crosslinker. The authors studied the binding properties of this novel material in comparison to EPI-dextran network polymers. It was capable of including iodine and several organic compounds, aniline, pyridine, benzaldehyde, butyric acid, *p*- and *o*-nitrophenol are a few examples. This study suggested that the inclusion ability could be useful in separation techniques based not only on separating molecules on the basis of their size but also of their shape, an example of which is the separation of *p*- from *o*-nitrophenol and of differences in their inclusion behavior [18]. This idea was further developed in the 1970s with the aim to prepare novel stationary phases for nucleic acids [19] and mandelic acid derivatives [20].

### 2.2. From the 1980s to the 1990s: Investigation on Polymer Properties and Applications

In the 1980s, research was more focused on exploring and understanding the properties of these new materials, rather than investigating only their binding abilities. In 1980, insoluble porous polymers such as polyurethane-CD network polymers were examined. They were obtained by using hexamethylene diisocyanate (HMDI), 1,3-bis (isocyanatomethyl) cyclohexane and 1,3-bis (isocyanatomethyl) benzene as crosslinkers. The synthesis consisted of stirring CDs with diisocyanate in pyridine subjected to heating. The properties of these new polymers were investigated more in depth compared to previous studies: thermal analysis, Brunauer, Emmett and Teller (BET) measurement of surface area, elemental analysis (detection of unreacted –OH groups in CD) and gas chromatography were employed to study the interactions between CD polymers and organic compounds (e.g., benzene, toluene, cyclohexane, ethanol, methyl ethyl ketone, propanol and others) [21]. Moreover, the influence of the type of crosslinker (1,2-ethanediol diglycidyl ether, 1,4-butanediol diglycidyl ether, 1,6-hexanediol diglycidyl ether, 1,3-butadiene diepoxide, 1,7-octadiene diepoxide, EPI) as well as the degree of crosslinking on guest binding properties of CDs were investigated [22].

In the 1980s and the 1990s, CD polymers were studied for food application for the first time. For example, grapefruit juice was debittered by removing naringin and limonin with the use of polymers, such CDs crosslinked with EPI, hexamethylene diisocyanate and phenyl isocyanate [23]. In addition, their encapsulation capability was also tested on other food components, such as caffeine, vanillin and theobromine [24].

Insoluble crosslinked polymers in the form of gels were examined for the sorption of textile dyes [25], and the same polymers were later used to entrap aromatic pollutants, such as phenol, *p*-nitrophenol, benzoic acid, *p*-nitrobenzoic acid, *β*-naphthol, chlorophenols and 4-*tert*-butylbenzoic acid [26].

### 2.3. 1999:“In the Beginning Was the Word”

The term “nanosponge” was used for the first time in 1999 by Min Ma and De Quan Li [27,28]. They described novel nanoporous polymers made up of CDs connected with diisocyanate linkers [28]. The preparation was very simple: CDs were mixed with the crosslinker in DMF and heated for 24 h. These NSs exhibited a surprisingly high adsorption capacity despite their low surface area, thus opening new possibilities in the water remediation field. Their properties, such as adsorption capacity, tunability and low cost, overcame the limits of the purification methods used so far, such as reverse osmosis and adsorption on activated carbon, or zeolites. A great advantage was the possibility to modify the properties of these polymers by changing the degree of crosslinking and the kind of crosslinker. The entrapment of organic pollutants from an aqueous medium was strictly dependent on the size of CD cavities, and the NSs were readily available after washing with an organic solvent.

The 20th century came to an end with an innovation in CD chemistry, which will influence a broad range of scientific fields. However, a decade passed before the importance of NSs was widely recognized.

### 2.4. From 2000 to 2009: New Millennium Came with New Applications

In the new millennium NSs found interesting opportunities in new fields. Nevertheless, already known applications of CD NSs were not neglected.

The problem of water purification was still one of the main points of interest, being a critical issue of daily life. The challenge consisted of improving water purification by developing novel insoluble CD materials with enhanced affinity to organic contaminants. A β-CD polymer highly crosslinked with EPI was prepared to entrap polycyclic aromatic hydrocarbons and pharmaceutical compounds from water [29]. The entrapment behavior showed the dependency on the polarity of the pollutant, being more effective with nonpolar compounds than ionized ones. The binding ability was affected by the presence of alcohols during the pollutant entrapment, as alcohols have a high affinity to CD cavity. Nevertheless, such competitive behavior offered an easy and efficient way to recover the polymer by washing in alcohol. Moreover the trapping potential could be enhanced via functionalization of CD NSs [30]. In 2005 (Italian patent), Trotta and collaborators replaced the potentially toxic diisocyanates used as NS crosslinking agents with carbonate compounds. They found that CDs crosslinked through carbonate bonds were in the form of micro- or nanoporous materials able to remove chlorinated persistent organic pollutants (POPs) from aqueous solutions even at low concentrations (such as a few ppb) and to strongly bind them [11,31,32].

The undesirable effects of some chemicals present in water even at very low concentrations created the need to improve filtration methods in order to discard them. Polyurethane cyclodextrin NSs were investigated for high-efficiency removal of aromatic and chlorinated hydrocarbons, using disinfection by-products with unpleasant odor as model pollutants. All the materials tested showed elimination of the pollutants to undetectable values; therefore, the efficacy obtained was higher than that of activated carbon [33]. Nanosponges were used also for discarding carcinogenic compounds from drinking water supplies, which can be harmful even at low concentrations, such as *N*-nitrosodimethylamine (NDMA). According to the study of Mhlongo et al., cyclodextrin polyurethane NSs showed great potential in NDMA entrapment [34].

CD NSs were not examined only for removing organic pollutants, but also for trapping heavy metals, thus finding application in environmental and pharmaceutical fields. The most common heavy-metal pollutants include arsenic, cadmium, chromium, copper, nickel, lead and mercury. The consumption of contaminated water causes the intake of these heavy-metals, which can accumulate in tissues and, therefore, are extremely harmful to human health. CDs crosslinked with pyromellitic dianhydride (PMDA) were tested for binding metal ions, such as Al (III), Mn (II), Co (II), Ni (II), Cu (II), Cd (II) and others. It was the first time that acidic properties of a CD polymer were investigated by pH-metric titrations with standard NaOH solutions in order to obtain protonation constant values of individual specific protogenic sites. The complexation of heavy metal cations on the polymer was tested at different pH values. In most of the cases the retention was mainly pH dependent and higher than 70% [35]. The idea was further developed by comparing different types of crosslinkers, such as naphthalene dianhydride and diphenyl carbonate (DPC) [36].

In the new millennium, nanotechnology and nanobiotechnology gained increasing importance and will change medicine greatly in the coming decades. Nanomedicine is believed to be a powerful tool to significantly improve the current existing drug delivery systems and treatments, thus improving the quality of life of patients.

Intense research on nanoparticulate systems began in this period, and various kinds of materials were proposed as drug delivery systems with improved solubility, pharmacokinetics, distribution, sustained release and cellular targeting of drugs.

CD NSs came to the scene due to their promising properties, which are attributable to their unique structure. At first, the research was focused on their capacity to encapsulate various kinds of drugs. Attention was also paid to their safety, negligible toxicity and biodegradability, as they were intended for human use. To assure the quality parameters listed above, efforts were made to better understand how nanosponges worked, their interaction with drugs and how they influenced the properties of the drugs. In addition to FTIR and HPLC methods, employed to examine the structure and loading properties, as well as DSC thermal analysis, X-ray diffraction, photon correlation spectroscopy, optical microscopy, TEM, electrophoretic mobility and zeta potential, hemolysis, cytotoxicity and in vitro release experiments were used and became essential to fully characterize these innovative drug delivery systems.

For the first time, Cavalli et al. tested the capacity of carbonate CD NSs to load drugs both lipophilic (e.g., dexamethasone or flurbiprofen) and hydrophilic (e.g., doxorubicin), and a sustained release of the drugs was achieved [2,37]. Carbonate CD NSs also showed the ability to significantly increase the solubility of the antifungal drug itraconazole and, thus, potentially improve its bioavailability. Moreover, the effect could be even enhanced by using additives such as copolyvidonum [38].

In the end of the decade, NSs found also application as a substrate for enzyme immobilization. Carbonate NSs increased the thermostability, pH stability and also storage stability of catechol 1,2-dioxygenase enzyme. NSs with this immobilized enzyme were used to create a small-scale bioreactor for “green” production of *cis*, *cis*-muconic acid, a precursor for industrially valued adipic acid [39].

### 2.5. From 2010 to 2015: Focus on Nanosponges as Delivery Systems

In these years, the capability of CD NSs to load molecules of pharmaceutical interest was extensively studied.

Alongside their use as drug carriers, they were found to be also suitable for gases, such as oxygen and carbon dioxide. The former can solve hypoxic conditions of tissues, the latter provide beneficial physiological effects like blood vessel dilation, blood circulation improvement and activation of gastrointestinal movement.

The capacity of cyclodextrins to store gases in their cavity has been known for a long time, dating back to 1987 when the encapsulation of carbon dioxide with CDs was patented in Japan anticipating its uses in cosmetics, cleansing and personal care products [40]. Among all, α-cyclodextrins were of choice because their gases have a low molecular weight and small size, unlike β-cyclodextrins, which have a higher dimension of the inner cavity and therefore do not fit the requirements to host gases. For the first time, Cavalli et al., and then Trotta et al., demonstrated that alongside cyclodextrins, crosslinked cyclodextrins, including β-CDs, were not only able to host gases but also turned out to be particularly advantageous in this field.

In particular, in 2010 Cavalli et al. [41] investigated the capacity of nanosponges synthesized using different kinds of cyclodextrins α, β, γ-CD crosslinked with 1,1′-carbonyldiimidazole (CDI) to deliver oxygen. These nanosponges, especially β-NS, were able to encapsulate, store and release oxygen for a prolonged period, which could be enhanced by means of ultrasound as external stimulus in an in vitro environment. The year after, Trotta et al. loaded inside carbonate β-CD NSs not only oxygen and carbon dioxide, but also 1-methylcyclopropene (1-MCP), opening a new possible application, such as the improvement of the vase life of *Dianthus caryophyllus* cut flowers. The oxygen loading and release confirmed the results of the study conducted by Cavalli et al., carbon dioxide was entrapped at atmospheric pressure and room temperature, and a significant amount of carbon dioxide was retained even at 373 K for 36 h under vacuum. 1-MCP included in β-CD NS showed a superior antiethylenic effect in long-lasting cut flowers in contrast to commercially available products [40]. Moreover, Seglie et al. demonstrated that 1-MCP encapsulated in β-CD NS was even more effective than the 1-MCP gaseous application at different concentrations, preventing pigment degradation in petals and reducing endogenous ethylene production [42]. Later, the effectiveness of the non-volatile formulation of 1-MCP complex in controlling *Botrytis cinerea* damage on carnation cut flowers was able to control fungal diseases of cut flowers in the postharvest environment [43].

A number of NSs-based drug delivery systems with different types of CDs and crosslinkers have been developed in these years. They contributed to improve the solubility, stability, sustained release, enhancement of permeability and bioavailability and activity of drugs. Moreover, they enabled alternative routes of administration to be chosen, thus favoring patient compliance and reducing side effects; ocular and transdermal delivery are a few examples.

Cancer drugs were extensively studied in order to find the best nanoparticulate delivery system capable of improving their efficacy and reducing their well-known side effects. CD NSs were proposed as a promising solution in the studies reviewed below.

Ansari et al. developed various β-CD NS crosslinked with diphenylcarbonate (DPH) with ratios 1:2, 1:4 and 1:8 to find the best carrier for loading paclitaxel [44]. Another attempt was made by Mognetti et al. who found an alternative to classical paclitaxel formulation in Cremophor EL: fluorescent NSs were synthesized and tested in vitro on cancer cells. As the anticancer activity of paclitaxel was enhanced, it was believed that the nanosponges adhering to/interacting with the cell membrane promoted the release of the drug [45].

Camptothecin, used for hematological and solid tumors, was encapsulated in DPH-linked NSs and tested on human prostate cancer cells. β-CD NS carriers were able to overcome chemical disadvantages of the drug and improve in vitro anti-tumor efficacy in androgen refractory models of prostate cancer DU145 and PC-3 [46].

Carboxylated β-CD NSs were effective nanocarriers for oral delivery of tamoxifen [47], for delivery of calcium in hyperphosphatemia [48], for curcumin in cancer treatment [49], naphthaleneacetic acid on rhizogenesis of globe artichoke [50] and acyclovir [51].

Lembo et al. evidenced another extremely powerful property of NSs: the possibility to make them fluorescent, which is particularly useful for cellular trafficking studies. The method consisted of adding a pre-formed carbonate NS to a fluorescein isothiocyanate solution in DMSO and incubating at 90 °C for 3 h. After the solid was recovered by filtration, it was reacted with succinic anhydride to obtain fluorescent NSs bearing carboxylic groups [51].

β-CD NS prepared with DPH as a cross-linker were successfully used for ocular delivery of dexamethasone. The drug was retained for a longer time in the eye, thus increasing its corneal permeability [52].

Nanosponges, as mentioned before, were employed also in drug delivery through the skin. Imiquimod used in the prevention and treatment of post-burn hypertrophic scars was loaded in β-CD/PMDA nanosponges [53]. The same kind of nanosponge was documented for the first time in Conte et al.’s study as a multifunctional ingredient in semisolid formulations for drug delivery to the skin [54]. The role of the NS in the solubilization and stabilization of benzoporphyrin-derivative monoacid ring A (BPDMA), all-trans retinoic acid (atRA) and on skin permeation of diclofenac (DIC) was tested. The nanosponge, being able to stabilize light-sensitive drugs and to localize the action of highly penetrating drugs in the external layers of skin, proved to be particularly useful in topical formulations.

Again, β-CD/PMDA nanosponges were investigated by Shende and co-workers to deliver meloxicam in order to improve its solubility and bioavailability as well as to prolong its release for anti-inflammatory and analgesic effects. Physical mixing, kneading and sonication were used to extend the duration of the drug release [55].

Positive results were achieved also with another non-steroidal anti-inflammatory drug (NSAID) such as ibuprofen. This drug was loaded inside β-CD/ethylenediaminetetraacetic acid (EDTA) dianhydride nanosponges. The dynamic properties of ibuprofen, especially in the gel state, were studied for advanced formulations using high-resolution magic angle spinning (HRMAS) NMR spectroscopy. The polymeric network was believed to affect the diffusive regimes of ibuprofen [56].

The administration of antioxidants is challenging due to their low solubility and physico-chemical stability. A number of studies were carried out to assess the suitability of NSs for the delivery of these molecules. CDI-linked β-CD NSs were used to deliver resveratrol and polyphenols found in apples (rutin, phloridzin and chlorogenic acid) [57]. The latter were successfully loaded in β-CD NS crosslinked with hexamethylene diisocyanate. Having significantly enhanced the photostability and antioxidant activities, NSs offered a potential drug delivery system for oral and topical delivery [58,59]. Moreover, gamma-oryzanol (GO), a mixture of ferulic acid esters, usually employed to stabilize food and pharmaceutical raw materials as sunscreen in cosmetic formulations, were loaded in NSs. The photodegradation of GO after exposure to UVA or UVB irradiation was slowed down, and the antioxidant effect was still present when included in nanosponges. In addition, a certain accumulation of GO was found in in vitro experiments on porcine ear skin [60].

Drug delivery is not the only field in which CD NSs found interesting opportunities: in these years agricultural and flame retardancy applications can be found.

Efforts were made to make agriculture more efficient, not only in terms of fertilization, but also to protect crops from pests and increase food shelf-life. Functionalized β-CD NSs were investigated for the growth, conservation, protection and disinfection of vegetable organisms [61]. NSs were used to increase the efficacy and bioavailability of nutrients and, thus, enhance the plant growth. Moreover, NSs may decrease toxic side-effects and enhance biodegradability of commercially available fertilizers. The positive effect of NSs as fertilizer carriers on plant growth was confirmed in a work focused on the development of a new iron fertilizer using a β-CD/PMDA nanosponge. The Fe-NS had a positive effect on the growth and re-greening in sweet corn as well as tomato plants [62].

As mentioned above, it is necessary not only to grow more plants, but also to protect the quality and safety of crops. The agricultural commodities might be devaluated by a number of pests, including fungi, after which contamination with harmful mycotoxins remains. Polyurethane NSs were studied to reduce the concentration of mycotoxins (Ochratoxin A) in aqueous solutions with positive results. CD NSs showed good potential for further use in decontamination of beverages produced from the affected commodities [63].

More and more emphasis is placed on the safety of materials in all fields, including electronics or construction. Polymers and textiles are very useful for preparation of cables, insulation, etc. However, the flammability of such materials is a serious problem limiting the application. To overcome this issue, materials are enriched with flame-retardant additives, such as phosphates, inorganic hydroxides, halogen and metal–halogen derivatives. These additives prevent ignition or slow down its development in three ways: endothermic degradation of retardant cooling down the substrate, creation of a protective char surface layer and/or emission of non-combustible gases. However, it is necessary to use a high amount of flame retardants (20–60 wt %) [64], which might affect the final properties of the materials. Development of efficient new fire retardant agents may lower the effective dose as well as preserve human health by using more eco-friendly compounds and eliminating the emission of toxic smoke [64,65,66].

Nanosponges were studied as novel flame retardant systems [64,65,66]. The first study was conducted by Alongi et al., consisting of nanosponges loaded with phosphorus derivatives, which were entrapped into the internal cavities of cyclodextrins and/or interstitial space between cyclodextrins and the crosslinker by mechanical grinding. Unlike traditional systems, this complex, which was stable in processing conditions, had the advantage of having NS acting as both carbon sources and foam forming agents able to generate phosphoric acid in situ directly, thus protecting the copolymer against combustion [64].

### 2.6. From 2016 to Present: State-of-the-Art and Future Prospects of Nanosponges

**Figure 1:**
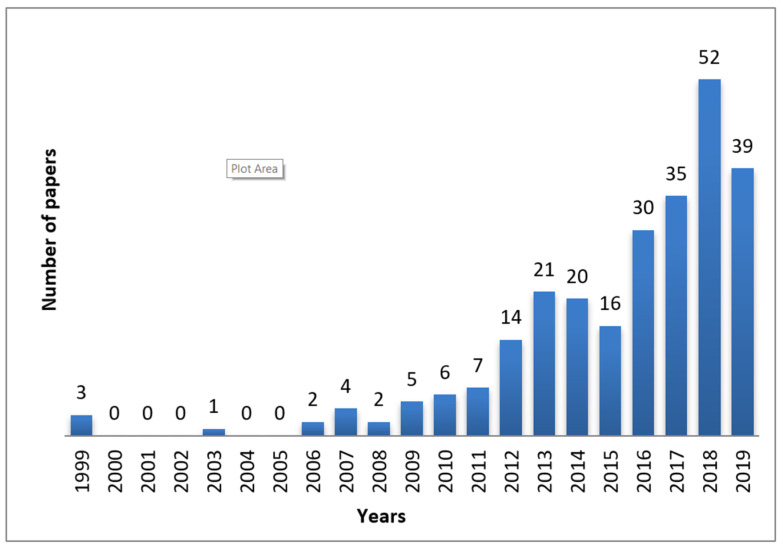
Number of papers on cyclodextrin-based nanosponges (CD NSs) published over the years from 1999 until 2019.

**Figure 2:**
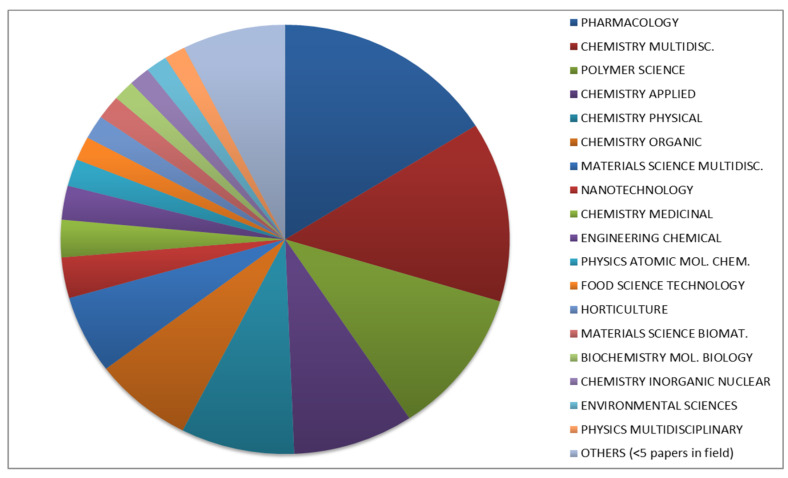
Research areas in which CD NSs are involved.

The increasing number of publications on CD-based NSs over the years shows that they have emerged during the last decade (Figure 1) and have attracted researchers’ attention worldwide in numerous fields (Figure 2).

The main area of investigation is nanomedicine [67], in which CD NSs have been employed mainly as drug delivery systems [68]. NSs gained great attention due to the ability to host various kinds of drugs, thus improving their bioavailability and, in addition to this, due to their lack of toxicity as demonstrated by Shende et al. in 2015 in their acute and repeated dose toxicity study [69]. 

In the last few years (2016–2019), all four CD-based nanosponge generations have been investigated in the pharmaceutical field [11]. β-CD crosslinked with DMC, CDI, DPC, PMDA and CA (citric acid), belonging to the first generation, have been employed in pharmaceutical research (Table 1). Some examples are provided below.

Patel and co-workers (2016) investigated the ability of β-cyclodextrin/DPC NSs to host both hydrophilic drugs, such as gemcitabine, and lipophilic drugs, such as bicalutamide, paclitaxel and letrozole. Lipophilic drugs had a higher drug loading capacity than hydrophilic ones because of their large number of lipophilic sites available for drug complexation. Moreover, the drug loading capacity seemed to be dependent on the synthetic route selected: it was lower if the NS was obtained by stirring than when a more powerful and intense method, like sonication, was used, a larger particle size being obtained in the former case. In the same year, β-CD/PMDA was used to encapsulate lansoprazole. The positive results obtained made this kind of NS promising for the treatment of gastric ulcers [72].

NSs were also used as multifunctional direct compression excipients for tablet designing without adding any binder, lubricant or disintegrant. This kind of NS was synthesized by crosslinking β-CD with CA [102].

In 2017, β-CD/CDI NSs loaded with anti-cancer drugs, such as erlotinib (an epidermal growth-factor receptor tyrosine kinase inhibitor) and camptothecin (an inhibitor of DNA Topoisomerase-I), increased their oral bioavailability, solubility and dissolution, minimizing the dose-related adverse effects [74,80,81]. Celecoxib, possessing analgesic and anti-inflammatory actions, was loaded in a β-CD/NN-methylene bisacrylamide nanosponge, which was incorporated in a hydrogel for topical application. The drug solubility and bioavailability were therefore enhanced [103].

β-CD/DPC NS was used for the delivery of drugs for the treatment of HIV, such as efavirenz and rilpivirine, in order to enhance their solubility and the bioavailability [83,84]. An improvement in the aqueous solubility was again achieved by using the same kind of NS (β-CD/DPC) for the delivery of chrysin, having antioxidant and anti-tumorous properties [78].

Curcumin, resveratrol and a combination of the two were loaded in β-CD NSs and tested [15,94,98]. Curcumin and resveratrol were combined in 2019 by Pushpalatha et al. in order to exploit their synergistic effect against breast cancer through transdermal delivery [15]. The nanosponges not only enhanced the release in vitro of both curcumin and resveratrol 10 and 2.5 times, respectively, but also the combination showed a synergistic cytotoxic effect on the breast cancer cell line selected. The year before, Pushpalatha and co-workers explored the effect of different kinds of crosslinked cyclodextrins (β-CD, DPC and β-CD, PMDA) for the delivery of resveratrol. Having selected the best crosslinker ratio, the NSs were compared in terms of physico-chemical characterization together with photodegradation, in vitro drug release, in vitro cytotoxicity and in vivo tests in order to find the most suitable choice for the selected drug [95]. Resveratrol and oxyresveratrol were also loaded in carbonate CD NSs to improve the solubility, release profile, photostability, antioxidant and cytotoxicity activity [104].

Alongside resveratrol, other natural antioxidants, such as ellagic and ferulic acids, were studied [86]. When loaded in NSs, their solubility was enhanced, making low solubility no longer a limit for their application in the food and pharmaceutical industries. Dhakar and co-workers studied the enhancement in aqueous solubility, antioxidant activity and in vitro cell toxicity of kynurenic acid (KYNA) loaded in β-CD/CDI NS. As a high solubilization and drug loading of KYNA were obtained, CD NSs proved to be suitable for biological applications [99].

Alongside drugs, the capability of NSs of reacting with fluorescent molecules was also explored, as this property would be particularly useful in vivo as they would act as fluorescent probes. This capability was confirmed by Ncube et al.’s study in which the isothiocyanate form of fluorescent dyes (rhodamine and fluorescein) was used to react with an epichlorohydrin β-CD polymer, found useful in cancer therapy [105]. Fluorescent NSs belong to the second generation of NSs, as reviewed by Caldera et al. [11].

The need to improve drug efficacy and minimize dosage in order to reduce side effects has stimulated the development of novel drug delivery systems capable of controlled release triggered by stimulating signals. These NSs, which belong to the third generation, have been developed recently. In 2016, Trotta et al. developed glutathione-responsive NSs capable of releasing entrapped anticancer drugs in response to intracellular stimuli, such as intracellular glutathione [75]. The synthesis consisted of a single-step reaction between β-CD and 2-hydroxyethyl disulfide in the presence of PMDA as a cross-linker [106]. GSH-NSs loaded with doxorubicin showed remarkably higher effectiveness than free drug in cancer cells characterized by high GSH content both in vitro and in vivo. In 2017, erlotinib hydrochloride, which was associated with severe toxicity when administered in a systemic and uncontrolled way, was loaded in GSH-NSs [85].

Biodistribution and in-vivo tumor growth inhibition studies revealed drug release to the cancer cell, thus preventing unnecessary drug exposure, and exhibited extended drug release proportional to the external GSH concentration. In 2018, plant hormones (strigolactones) were loaded into GSH/pH-sensitive NSs in order to investigate whether this stimuli-responsive nanocarrier reduced the viability of prostate cancer cells in vitro. The cytotoxic effects on prostate cancer cells have been enhanced after incorporation of SLs into GSH/pH-sensitive NSs [107].

Other types of pH-sensitive NSs were also explored. In 2019, cyclodextrins and calixarenes were combined to develop pH-sensitive NSs (CyCaNS), the adsorption and release capabilities of which were tested using as a model drug for tetracycline antibiotics [108]. pH sensitivity, according to Fontana et al., may be due to the Coulomb interactions between the positively charged guest molecule and the average charge density on the polymeric framework (due to the presence of ionizable amine or carboxyl groups deriving from chemical post-modification). Interesting improvements of the biocidal activity were achieved. These improvements may be attributable once again to the presence of particularly favorable Coulomb interactions occurring between the NS carrier and the bacterial cell wall. Further studies are ongoing in order to better understand the possible mechanisms implicated in the CyCaNS-bioactive molecule-bacterial cell interaction.

NSs obtained via interaction between template molecules and polymers by covalent, semi-covalent or non-covalent bonding are included within the group of Molecularly Imprinted Polymers (MIPs) and belong to the fourth generation [11]. The product is a heteropolymer matrix with specific recognition elements for the template molecule, even though it has been removed. MIPs find applications in several fields alongside drug delivery, such as separation sciences and purification, biological antibodies receptors systems and catalysis. Deshmukh and co-workers developed biomimetics for glucose estimation using molecularly and non-molecularly imprinted polymers of β-CD/PMDA NSs. The rapid extraction of the template from the samples, high adsorption efficiency and non-toxicity are some of the properties of MIP-NSs. They have several advantages over non-molecularly imprinted polymers (NIP NSs) due to the diffusion of the template in the formed cavity and its high surface area.

Trotta and co-workers synthesized new MIP-NSs by cross-linking β-CD with CDI in DMF in the presence of L-DOPA as a template molecule (a pro-drug for the treatment of Parkinson’s disease). It has been confirmed that MIP-NSs show a slower and more prolonged release profile making them a promising alternative for storage and controlled delivery of L-DOPA [109]. MIPs could be also an effective approach for the synthesis of catalysts capable of overcoming limitations for processes requiring an optimal arrangement of several reacting molecules or of converting bulky molecules in two-phase systems [110,111,112,113,114].

In 2018, attempts were made to overcome the inherent lack of cellular binding ability of NSs, which has limited their application in drug delivery. In fact, medical research has recently invested heavily on drug delivery systems capable of reaching the target site (cell/tissue/organ) in order to improve the efficacy of the drug and limit its adverse effects. This therapeutic approach called “targeted drug delivery” has been experimented especially on anticancer drugs to limit their toxicity. Various approaches have been proposed, including the functionalization of nanoparticles with natural ligands, which bind to specific receptors on the surface of the target cells, thus increasing cellular drug uptake [12]. CD-based NSs, even if still at an early stage, have been studied in this field of application as they can be easily functionalized with ligands as mentioned in the introduction of this review. A method explored successfully by Singh P. and co-workers was the functionalization of the surface of CD-based NSs with cholesterol, which is a ubiquitous endogenous molecule, responsible for cell interactions and protein binding. Doxorubicin was selected as a model drug, and its cellular uptake revealed an enhanced effect of doxorubicin when loaded in this innovative carrier [92].

This approach was also used for theranostic nanomedicines, which have attracted huge interest for imaging-guided drug/gene delivery in cancer treatment because of the combination of diagnostic and therapeutic functions. For example, folic acid was used as a targeting agent in a novel theranostic system based on a CD NS polymer anchored on the surface of magnetite nanoparticles, which was then decorated with folic acid [97]. Facile condensation polymerization with carboxyl-functionalized nitrogen-doped carbon-quantum dots (CQDs) and β-cyclodextrin as multifunctional monomers has been used to synthesize fluorescent hyper-crosslinked β-CD–CQD hybrid NSs for tumor theranostic application. Doxorubicin (DOX) was loaded to β-CD–CQD via host–guest complexation, showing a pH-responsive, controlled release in the stimulated tumor microenvironment. Their potential application in tumor imaging has been demonstrated by easy synthesis, excellent biocompatibility, strong bright blue fluorescence emission, pH-responsive sustained release and enhanced anticancer activity [115].

Alongside drugs, mosquito repellents were also encapsulated inside nanosponges to improve the performance of *N*,*N*-diethyl-meta-toluamide (DEET) commonly used to prevent dangerous infections transmitted by insects. DEET itself shows repellence against mosquitoes, bugs and ticks but is not effective for a long time, whereas when encapsulated inside CD NSs it has a prolonged residence time. DEET-loaded NSs were synthesized using CDI and PMDA. DEET was complexed with the β-CD cavities before or after cross-linking. The encapsulation efficiency, loading capacity and washing durability were studied. The persistence of DEET on polyester fabrics was observed in CDI-NSs preloaded with 5:1 molar ratio of β-CD due to stronger DEET–NS interaction [116]. Moreover, DEET was loaded in β-CD/PMDA NS microfibers, and its release over time was monitored. Cecone and co-workers produced these fibers using an electrospinning process, and from their pyrolysis a novel carbon material was obtained. There has been tremendous progress with electrospun fibers because of their various applications such as filtration, tissue engineering, biosensors, drug delivery, wound dressings and enzyme immobilization [117,118].

Alongside pharmaceutical application, in the last few years NSs have been studied also in other fields, of which the food industry, environment, textile industry, solid-phase extraction and catalysis (Table 2) are a few examples.

The increased consumption of packaged food and consumer demand for safer and minimally processed food have driven research towards natural alternatives to synthetic antimicrobials and active/intelligent packaging capable of releasing these antimicrobials. NSs have been investigated as feasible candidates due to their encapsulation ability, sustained release, low cost and lack of toxicity. In the last few years the antimicrobial properties of cinnamon and coriander essential oils have been exploited by incorporating them into CD NSs to successfully create stable controlled release systems [123,124]. In the study conducted by Silva et al., various kinds of NSs (synthesized using CD monomers, such as α-CD, hydroxypropyl-β-CD and maltodextrin) were investigated in order to find the most suitable ones for both essential oils. The results demonstrated that loading was dependent on the type of solvent used and that NSs had different affinities for the oils tested as well as for each of their components [122].

The problem of water pollution is still a significant concern, although a lot of effort has been made by the industrial sector over the last 30 years. In 2017, Salgin et al. tested the capability of NSs (β-CD/HDMI) to remove organic pollutants such as p-nitrophenol (p-NP) from water [125]. Still, in 2019 the traditional use of NSs is proposed again for the removal of pollutants such as heavy metals [126], boric acid and organic micropollutants [127]. Rubin Pedrazzo and co-workers demonstrated that nature-friendly NSs obtained by crosslinking β-CD with CA in water were also capable of adsorbing a high amount of heavy metal ions. Particularly new (there is little information about it in the literature) is the use of NSs as advanced pharmaceutical carriers combined with textile materials, namely bio-functional textiles [128]. They are promising as wearable platforms for controlled drug release through skin [128,129]. In the study conducted by Mihailiasa et al., cotton fibers were functionalized with melatonin-loaded carbonate NSs (β-CD/CDI) to prepare a bioactive functional fabric. Melatonin formed a molecular dispersion in the NS cavities, and its loading efficiency was estimated at 8 wt %. An in vitro release study was conducted and showed that the melatonin release rate from the functionalized fabrics could be described by a zero-order law [129].

NSs have also aroused great interest for green extractions through solid-phase extraction. They have already been studied for extracting steroids from a complex matrix like urine, using both β-CD-EPI NS and β-CD urethane NSs [130]. The latter has also been used as a sorbent of carcinogenic aromatic amines from water [130]. These NSs have a huge potential because they are inexpensive, versatile and have evolved in order to make the extraction procedure greener and have a better performance. Thus, efforts are being made not only to prepare non-polluting and re-utilizable NSs, but also functionalized ones with both ionic and neutral groups to adsorb organic compounds, metals and ions simultaneously, as well as amphoteric NSs to be used over a wide pH range.

Another research field in which NSs, especially the carbonate ones, have been investigated is catalysis. Sadjadi and co-workers suggested CD NS-based catalysts as potential candidates for promoting chemical reactions. Heteropolyacids (HPAs) are considered a non-toxic class of catalysts and have been widely used for their strong Bronsted acidity and redox potential. The NS was amine-functionalized followed by HPA immobilization. Synthesis of a variety of xanthenes in aqueous media has been carried out using this catalyst. The merits of this novel procedure were the green and mild reaction conditions as well as excellent yield. Another efficient hybrid catalyst was developed combining HPAs with ionic liquids (ILs). HPA-IL immobilized in the cavities of CD NS was used to promote the cascade reaction of hydrazine hydrate, ethyl acetoacetate, α- or β-naphthol and benzaldehyde. In terms of reactivity and reusability, both hybrid catalysts showed superior catalytic performance [121,131].

Considering the great variety of applications, researchers are increasingly aware of NS potential and need to fully characterize them in order to understand their structure and mechanism of action. To study the interaction of drugs loaded with NS and to emphasize the process of their fabrication, synthesis and design, NS characterization using suitable analytical techniques is the need of the hour. Full analytical characterization of NSs can be useful to select the most appropriate polymer, crosslinker and increase the pharmaceutical applications of these systems along with possible patenting and marketing implications [132].

Knowledge of the analytical tools can significantly improve the assessment of the quality parameters of NSs, i.e., their safety, negligible toxicity, superior inclusion capability, marked swelling behavior and biodegradability, which are fundamental for their use in drug delivery, drug targeting, tissue engineering and regenerative medicine. Therefore, with the growing importance of CD NSs, many characterization techniques have been used. Fourier transform infrared spectroscopy in attenuated total reflectance geometry (FTIR-ATR), CHNS/O analysis, scanning electron microscopy (SEM), energy dispersive X-ray spectroscopy (EDX) analysis, light scattering analysis [133], low-frequency Raman scattering [134], high-resolution magic angle spinning (HRMAS) NMR spectroscopy, carbon-13 nuclear magnetic resonance (^13^C NMR), proton nuclear magnetic resonance (^1^H NMR), thermogravimetric analysis (TGA), potentiometric titration, Brunauer–Emmett–Teller (BET) analysis, transmission electron microscopy (TEM), differential scanning calorimetry (DSC) and X-ray diffraction (XRD) have been extensively employed in the studies over the years. In addition, the nano-formulations developed need to be evaluated as far as encapsulation efficiency and loading capacity, stability, in vitro drug release, in vivo pharmacokinetic release, efficacy and toxicity are concerned in order to ensure quality and efficacy [35,37,53,56,57,73,76,78,98,109,122,125,135,136,137,138,139,140].

In recent studies, special attention has been given to optimizing formulations in order to find the best solution for the active molecule to be delivered, also by means of mathematical tools, such as a design approach. Experimental design helps to minimize the experiments, develop the process and improve the product quality. It consists of exploring the behavior of NSs in various experimental conditions in a limited number of tests and straightforward identification of the variables that affect the system the most, taking into account the synthesis [141] or the formulation process [14,15,16]. For example, this approach was adopted by Kamble and co-workers to synthesize NSs considering the effect that various levels of crosslinking agents and βCD concentrations had on porosity, drug encapsulation, zeta potential and drug release [17]. Furthermore, Pushpalatha and co-workers optimized the curcumin-resveratrol loaded NS hydrogel formulation using a factor 3-level Box–Behnken design. The concentrations of the two ingredients used for the hydrogel, together with the pH, were selected as independent variables at three levels. Transdermal flux of curcumin, transdermal flux of resveratrol and spreadability of gel were the dependent variables evaluated for optimization [15]. Singireddy and co-workers optimized the synthesis of CD NSs to avoid low yields and batch-to-batch variations due to differences in experimental conditions (reaction temperature in °C, reaction time in min and stirring speed in rpm). The reaction conditions for the synthesis of NSs were optimized by using central composite design and response surface methodology [141].

**Figure 3:**
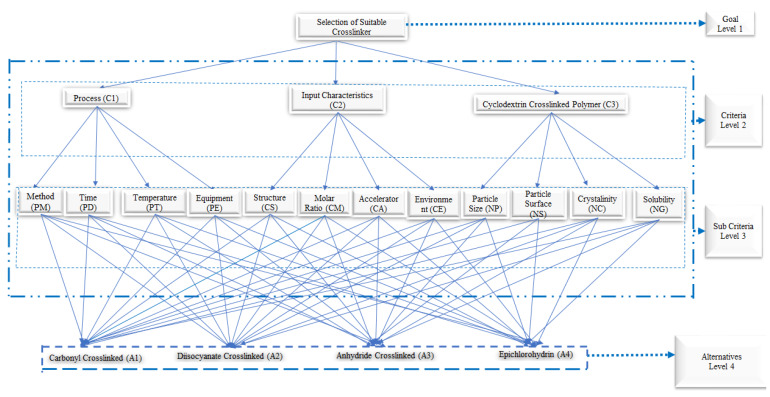
Hierarchy analysis approach to choose the best cross-linker.

Pushpalatha and co-workers also used the hierarchy analysis approach to choose the best cross-linker among various kinds for obtaining NSs for drug delivery [14]. The most common cross-linkers were investigated. The selection was made on the basis of the process, materials and physicochemical characteristics of the output following the schematic procedure present in Figure 3. From this study, it emerged that this kind of analysis helped to reduce the number of experiments, shorten the development process and improve product quality.

## 3. Conclusions and Future Perspectives

**Figure 4:**
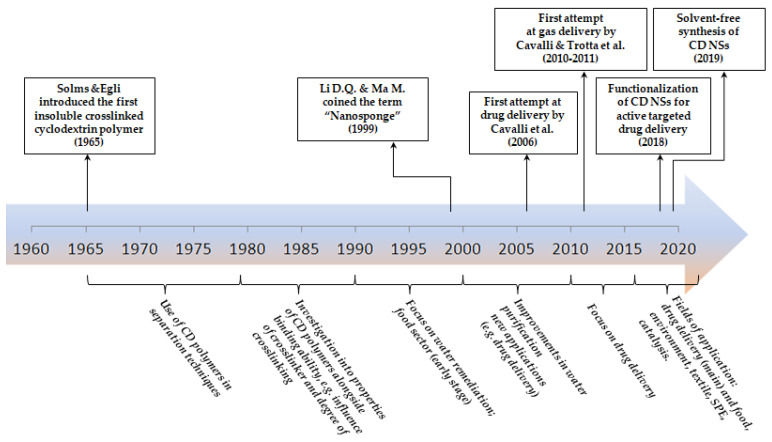
Timeline of the historical development of cyclodextrin-based nanosponges.

The historical description of CD NSs shows how they emerged over the years (Figure 4, Table 1 and Table 2).

It all began in the 1960s when simple network polymers made up of crosslinked CDs were introduced for the first time. Their binding properties tested on organic compounds suggested a possible application in separation techniques, which was further developed in the 1970s with the production of stationary phases for nucleic acids, etc.

In the 1980s, research explored new polymers and made efforts to understand their properties alongside their binding ability. The influence of the crosslinker and the degree of crosslinking on guest binding properties of CDs were investigated for the first time.

In the 1990s, CD polymers found application as debittering agents and food component carriers (e.g., caffeine, vanillin and theobromine). Moreover, in the water remediation field, they overcame the limits of purification methods used up to then due to their high adsorption capacity, tunability and low cost. At the end of this decade they were called “nanosponges” for their nanoporous sponge-like structure.

In the new millennium, new opportunities for nanosponges were studied without neglecting the known applications, such as water purification, with efforts made to improve NS trapping potential by functionalizing them or by replacing potentially toxic crosslinking agents with carbonate compounds, improving the removal of organic pollutants even at a few ppb.

For the first time, nanosponges were investigated as drug delivery systems. Different kinds of drugs were successfully loaded, and a sustained release was achieved. In addition, their safety, negligible toxicity and biodegradability became a matter of concern as they were intended for human use.

This field of application was extensively studied in the following years (2010–2015) lengthening the list of drugs delivered, e.g., anticancer drugs, polyphenols, L-Dopa, NSAIDs and gases of pharmaceutical interest (i.e., oxygen and carbon dioxide).

The period 2016–2019 has seen pharmaceutics as the main field of application. All NS generations are present, including innovative smart nanosponges capable of releasing drugs triggered by external stimuli (i.e., pH and GSH) and the most recent ones having natural ligands grafted on the surface able to perform active targeting.

In these years, alongside the pharmaceutical field, the great versatility of NSs has been confirmed by studies conducted in the food industry identifying new applications, such as active/intelligent packaging. Other applications have been found in the environmental field, the textile industry, solid-phase extraction and catalysis.

Much attention is being paid to fully characterize NSs in terms of structure and mechanism of action with a view to selecting the most appropriate polymer also with the aid of mathematical tools, such as a design approach to rationalize experimentation and improve the product quality.

The success of cyclodextrin-based nanosponges certainly is due to their ability to keep up with the times while retaining their initial features, i.e., low cost, environmental compatibility, non-toxicity and the ability to host various kinds of molecules. Their synthesis has evolved in the direction of greener processes culminating in the most recent solvent-free synthesis. All of these advantages would make NSs suitable for future industrial scale up.

For the reasons discussed above, NS research has not yet reached its conclusion. On the contrary, the potential advantages that could be obtained from their use certainly justify further studies aimed, on the one hand, at investigating in greater depth their existing fields of application in which appropriately optimized NSs are useful as carriers and, on the other, at exploring new fields in which their potential could be exploited to the full as a promising, safe innovation for human health and activities.

## Figures and Tables

**Table 1:** Historical excursus of publications on CD-based NSs employed in pharmaceutical research.

Year	Nanosponge/Cross-Linker	Drug	Indication	References
2010–2015	β-CD/DPC	Camptothecin	Pharmaceutical application	[46,70]
	β-CD/CDI	Resveratrol	Pharmaceutical application	[58]
	Fluorescent NS	Paclitaxel	Pharmaceutical application	[45]
	Carboxylated nanosponges	Acyclovir	Antivirial efficacy	[51]
	β-CD/CDI	Rutin, phloridzin and chlorogenic acid	Anti-cancer, anti-diabetic, antiobesity, neuronal protective properties etc.	[57]
	β-CD/DPC	Gabapentin	Treatment of partial seizures in pediatric	[71]
2016	β-CD/PMDA	Lansoprazole	Gastric ulcers	[72]
	β-CD/DPC	Quercetin	Cancer treatment	[73]
	β-CD /CDI	Erlotinib hydrochloride (ERL)	Cancer treatment	[74]
	GSH–NS (β-CD/PMDA)	Doxorubicin	Anticancer activity	[75]
2017	β-CD/EDTA	Ibuprofen	diseases	[76]
	β-CD/DPC	2-(3,4-dimethoxyphenyl)-3-phenyl-4H-pyrido [1,2-a] pyrimidin-4-one (DB103)	Cardiovascular diseases, drug-eluting stents (DES)	[77]
	β-CD/DPC	Chrysin	Optimal anti-oxidant and anti-tumorous properties	[78]
	β-CD/DPC	Nifedipine	Treatment of angina pectoris and hypertension	[79]
	β-CD/CDI	Camptothecin (CPT)	Antitumor efficacy, inhibition effect on prostate cancer	[80,81]
	β-CD/DPC	Cefadroxil (CFD)	Against variety of Gram-positive and Gram-negative bacteria	[82]
	β-CD/DPC	Efavirenz and Rilpivirine HCl	HIV	[83,84]
	β-CD/PMDA	Erlotinib glutathione	Cancer	[85]
2018	β-CD/DMC	Ellagic acid	Cancer	[86]
	β-CD/DPC	Mebendazole	Lymphatic worm infestations	[16]
	β-CD/TDI	Naproxen	Inflammation	[87]
	Electrospun	*N*,*N*-diethyl-3-toluamide	Infectious diseases	[88]
	β-CD/PMDA			
	β-CD/CDI; PMDA	Rilpivrine	HIV	[89]
	β-CD/CDI	Atorvastatin calcium	Dyslipidemia	[90]
	β-CD/DPC	Norfloxacin	Urinary tract infections	[91]
	β-CD/CDI	Doxorubicin	Cancer	[92]
2019	β-CD/NDCA	Sage essential oil	Diabetes	[93]
	β-CD/DPC; PMDA	Curcumin and Resveratrol	Cancer	[15,94,95]
	β-CD / DPC	Ferulic acid and Imatinib Mesylate	Cancer	[17,96]
	β-CD/EPI	Curcumin	Cancer	[97,98]
	β-CD/CDI	Kynurenic acid and Paliperidone	Neurological disorders and Schizophrenia	[99,100]
	β-CD/PMDA	Imiquimod	Topical diseases	[101]

**Table 2:** Historical excursus of publications on CD-based NSs employed in food, textile, environmental fields, etc.

Years	Nanosponge/Crosslinker	Adsorbate	Field	References
1980–2000	CDs/HMDI, phenyl isocyanate, EPI	Naringin, limonin, debittering of grapefruit juice,	Food	[23]
	CDs/EPI	Caffeine, vanillin and theobromine		[24]
	CDs / EPI, HMDI and Phenyl isocyanate	Textile dyes	Textile	[25]
		Aromatic pollutants, such as phenol, *p*-nitrophenol, benzoic acid, *p*-nitrobenzoic acid, *β*-naphthol, chlorophenols and 4-*tert*-butylbenzoic acid	Environment	[26]
	CDs/HMDI or toluene 2,6-diisocyanate	Organic pollutants	Water Treatment (Environment)	[119]
2000–2009	β-CD/EPI	Naphthalene, 2-naphthol and naproxen	Water Treatment (Environment)	[29]
	CDs/CDI, DMC	Chlorinated persistent organic pollutants (POPs)	Water Treatment (Environment)	[32]
	CD/HMDI or toluene-2,4-diisocyanate	p-nitrophenol and pentachlorophenol,		[30]
	β-CD/PMDA	Heavy metals (Al (III), Mn (II), Co (II), Ni (II), Cu (II), Cd (II) etc.)	Water Treatment (Environment)	[35]
		Carcinogenic N-nitrosodimethylamine (NDMA)	Water Treatment (Environment)	[34]
	Carbonate NSs	Increase the thermostability, pH stability and storage stability of catechol 1,2-dioxygenase	As a substrate for enzyme immobilization	[39]
2010–2019	β-CD/DPC	Phosphorus derivatives	Flame retardancy	[64]
	α, β, γ-CD / CDI; PMDA	Oxygen	Hypoxia/Reoxygenation and Biomedical application	[41,120]
	β-CD /CDI	Oxygen, carbon dioxide and 1-methylcyclopropene 1-MCP carriers.	biomedical, environmental and floriculture application	[40]
	β-CD/PMDA	Iron fertilizer	Agricultural application	[62]
	CD/DPC	Xanthene derivatives	Catalytic Activity	[121]
	CD/CDI	Coriander essential oil	food packaging	[122]
